# Supplementary material for: Sarcoma classification by DNA methylation profiling
Source: Nat Commun. 2021 Jan 21;12:498. doi: 10.1038/s41467-020-20603-4 (PMC7819999; doi:10.1038/s41467-020-20603-4)
Supplement: Supplementary file 1 — Supplementary Information [file 41467_2020_20603_MOESM1_ESM.pdf]

Supplementary Figure 1

a

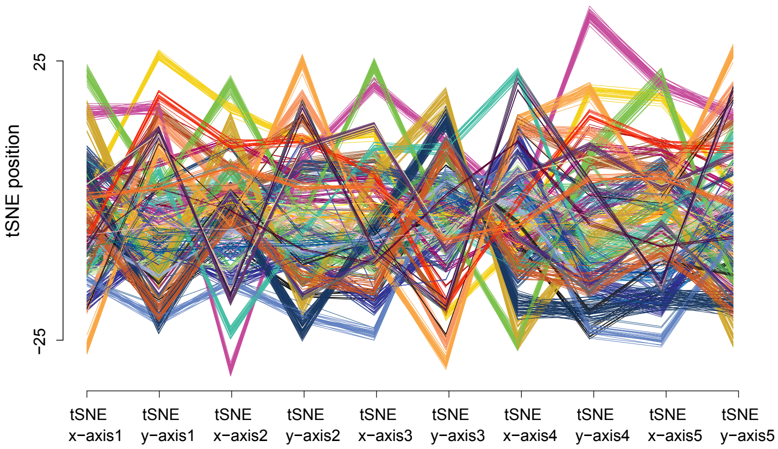

b

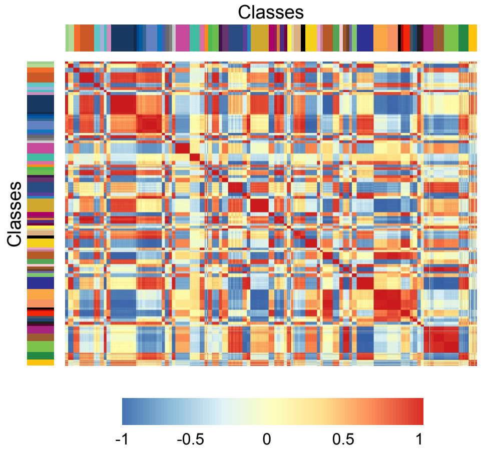

Supplementary Figure 2

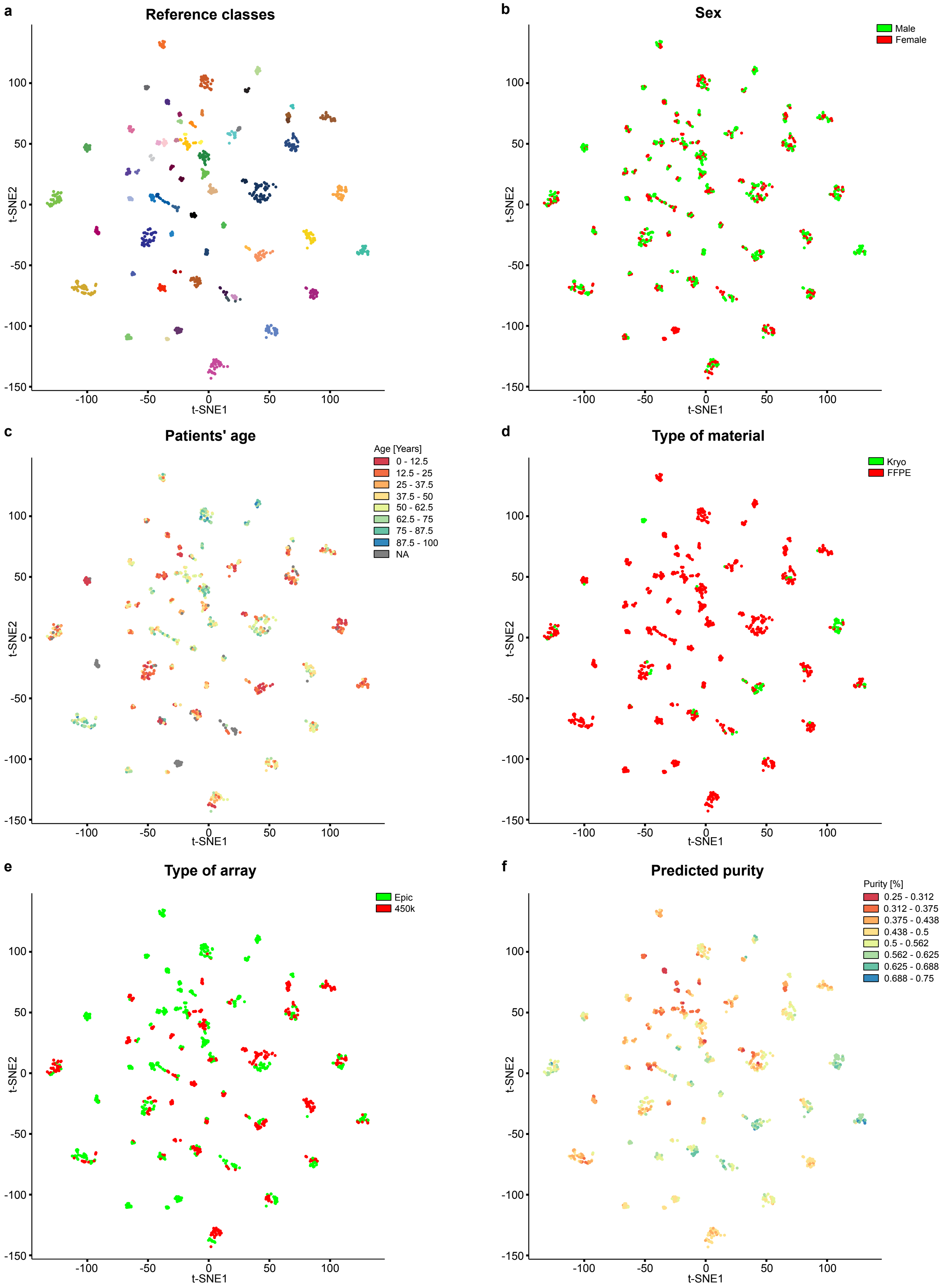

Supplementary Figure 3

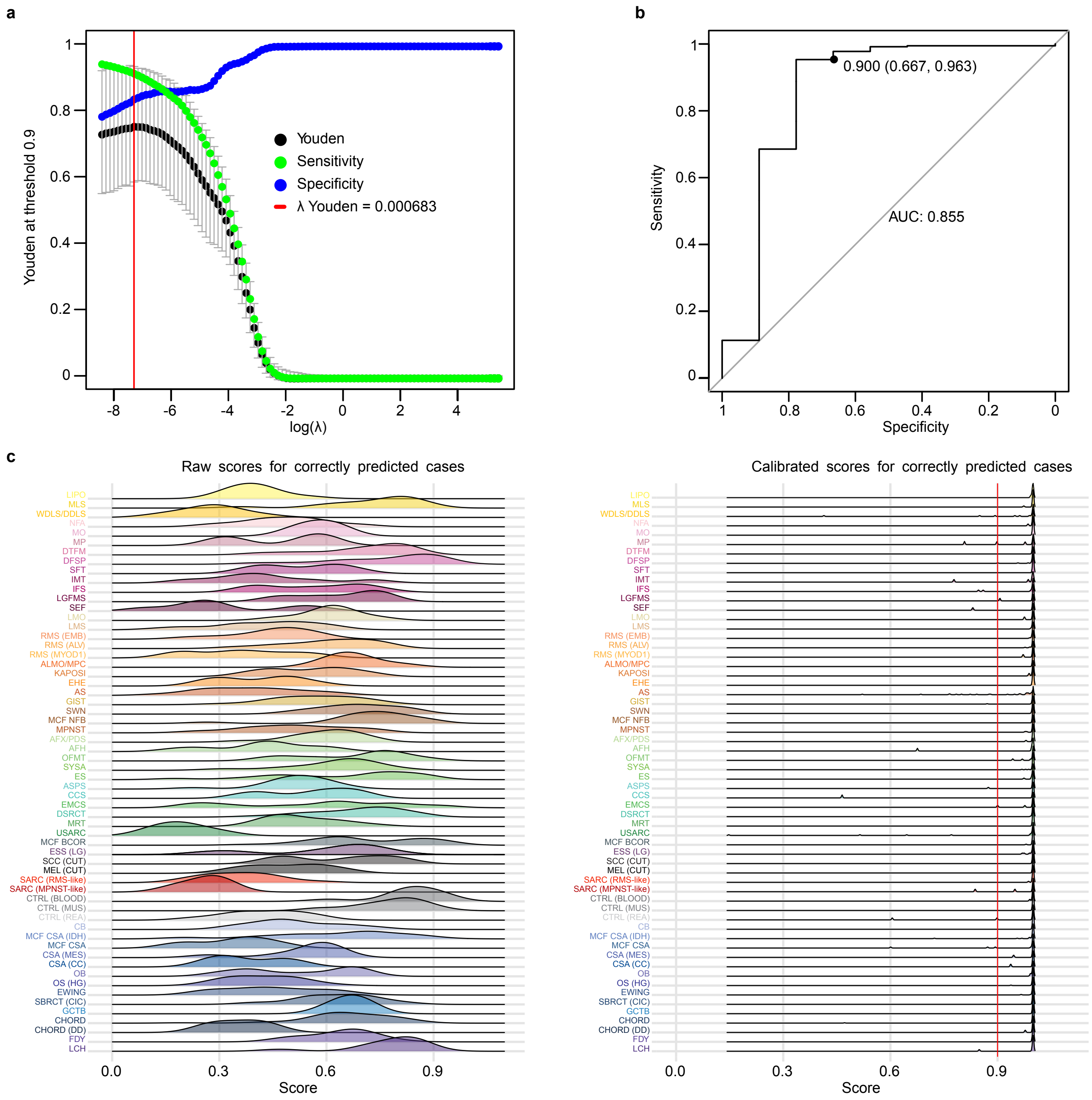

Supplementary Figure 4

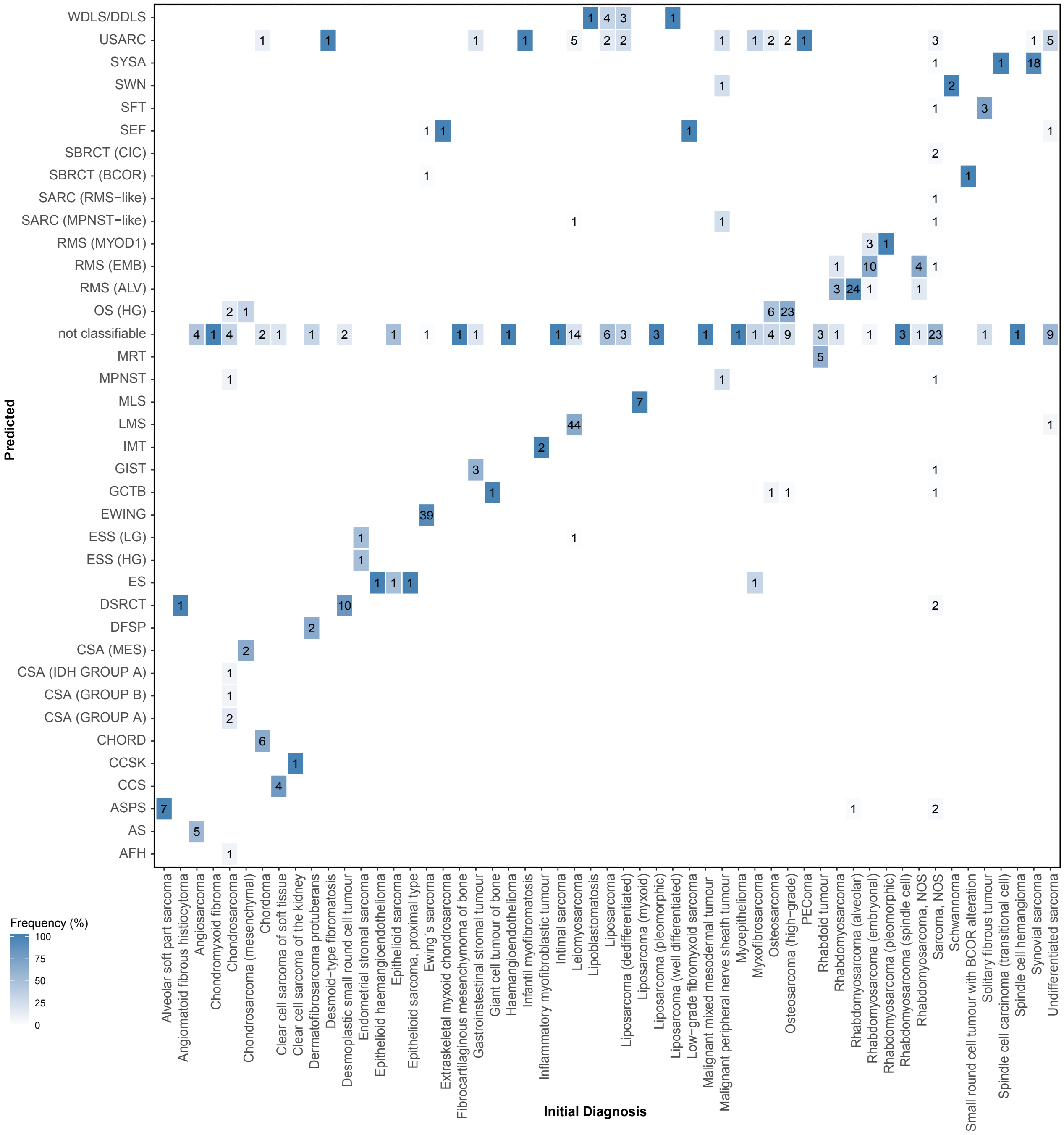

Supplementary Figure 5

Angiomatoid fibrous histiocytoma (n = 8)

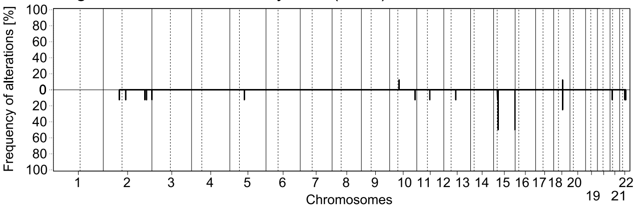

Atypical fibroxanthoma and pleomorphic dermal sarcoma (n = 14)

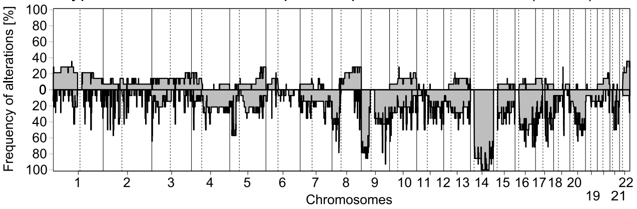

Angioleiomyoma and myopericytoma (n = 16)

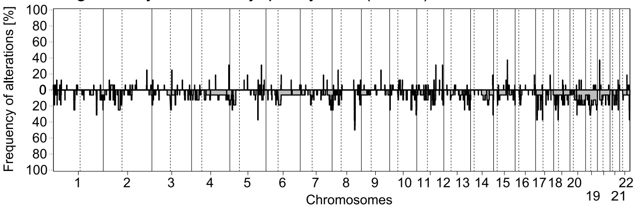

Angiosarcoma (n = 37)

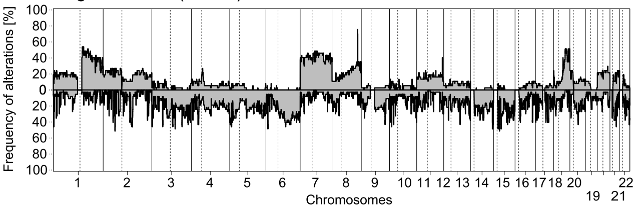

Alveolar soft part sarcoma (n = 16)

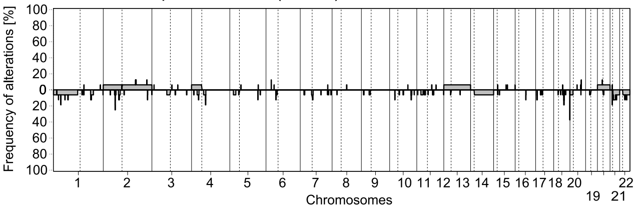

Chondroblastoma (n = 10)

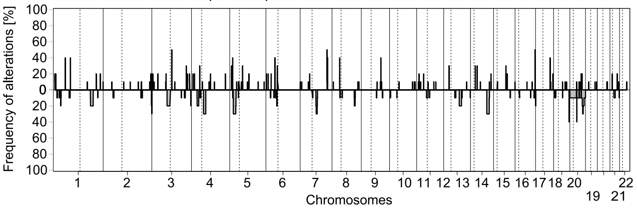

Clear cell sarcoma of soft parts (n = 7)

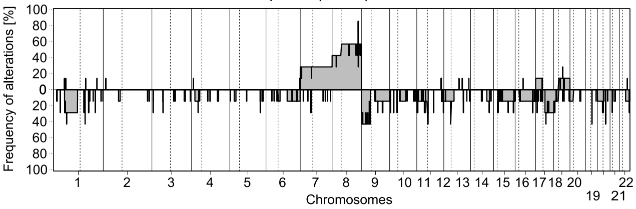

Clear cell sarcoma of the kidney (n = 11)

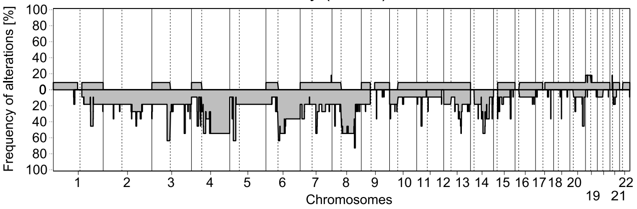

Chordoma (n = 59)

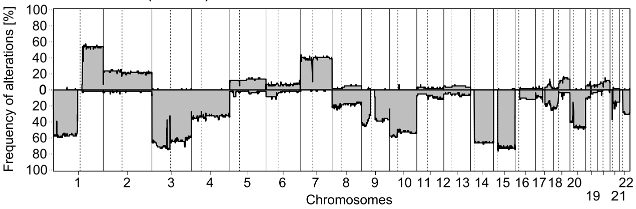

Dedifferentiated chordoma (n = 8)

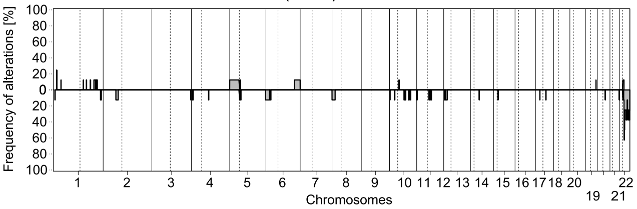

Control - blood (n = 10)

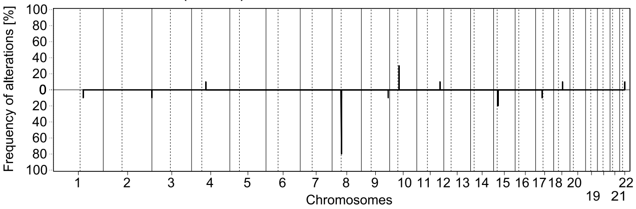

Control - skeletal muscle tissue (n = 8)

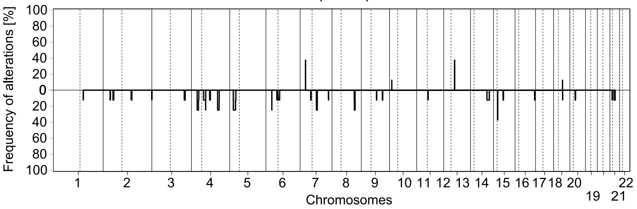

Control - reactive tissue (n = 10)

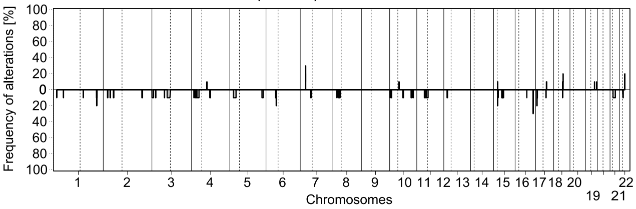

Chondrosarcoma - clear cell (n = 7)

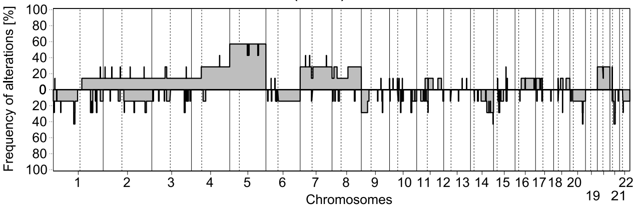

Supplementary Figure 5 - page 2

Conventional chondrosarcoma - group A (n = 9)

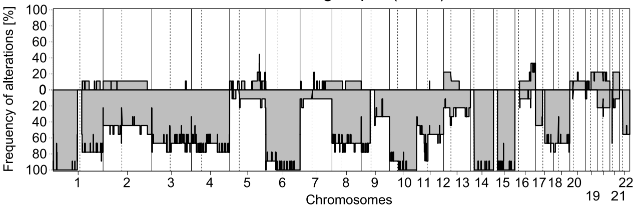

Conventional chondrosarcoma - group B (n = 9)

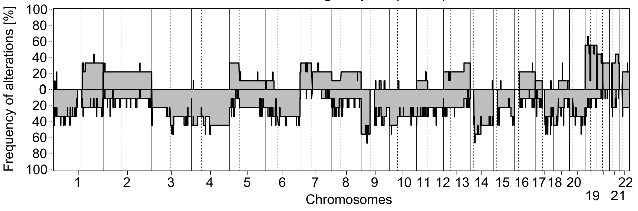

Conventional chondrosarcoma - IDH group A (n = 29)

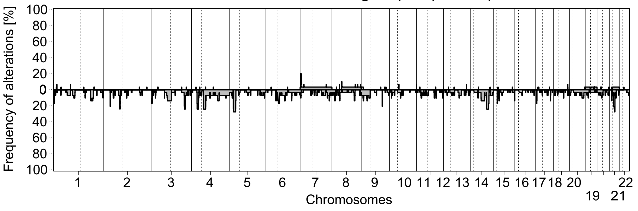

Conventional chondrosarcoma - IDH group B (n = 13)

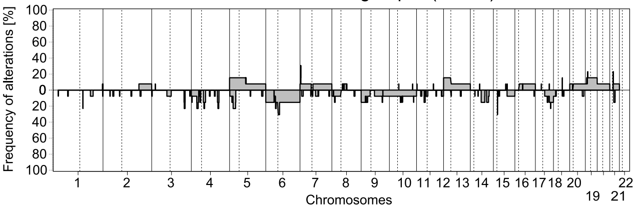

Chondrosarcoma - mesenchymal (n = 8)

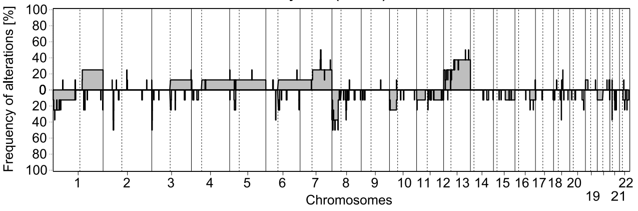

Dermatofibrosarcoma protuberans (n = 37)

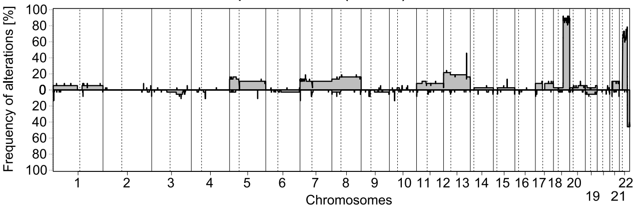

Desmoplastic small round cell tumour (n = 26)

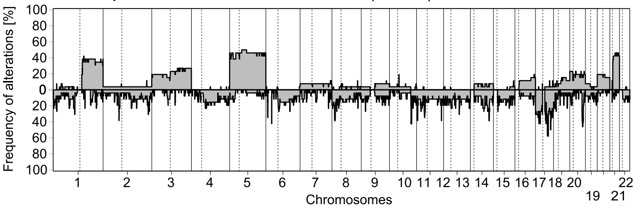

Desmoid-type fibromatosis (n = 13)

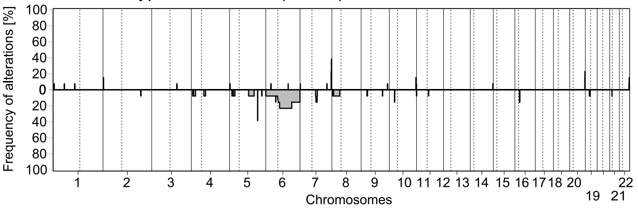

Extraskeletal myxoid chondrosarcoma (n = 10)

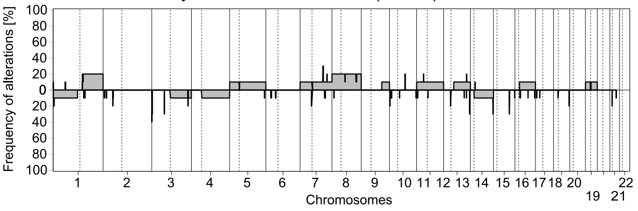

Epithelioid hemangioendothelioma (n = 9)

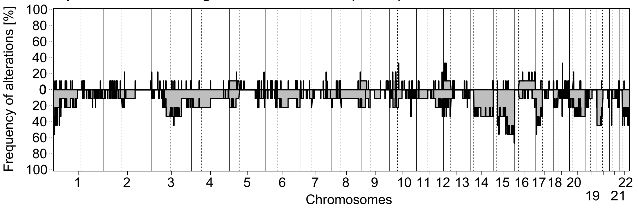

Epithelioid sarcoma (n = 18)

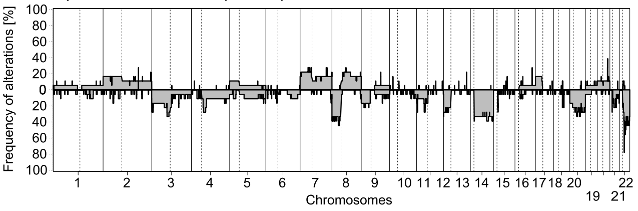

Endometrial stromal sarcoma - low grade (n = 16)

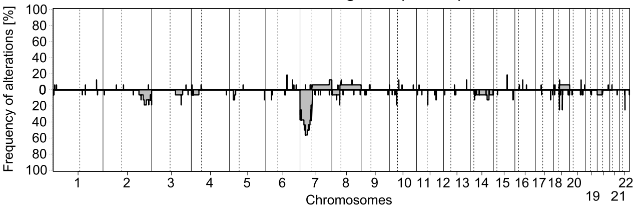

Endometrial stromal sarcoma - high grade (n = 9)

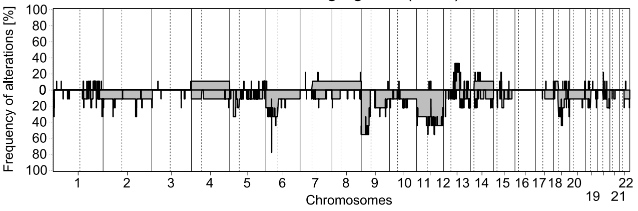

Ewing's sarcoma (n = 37)

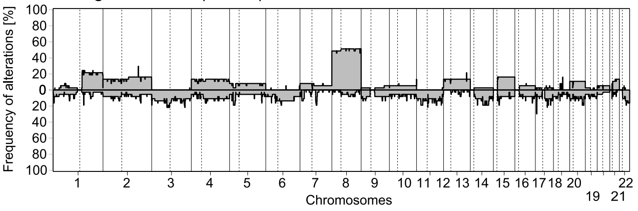

Supplementary Figure 5 - page 3

Fibrous dysplasia (n = 12)

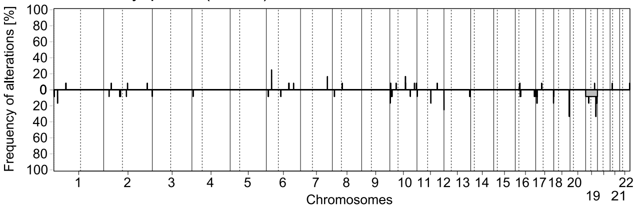

Giant cell tumour of bone (n = 9)

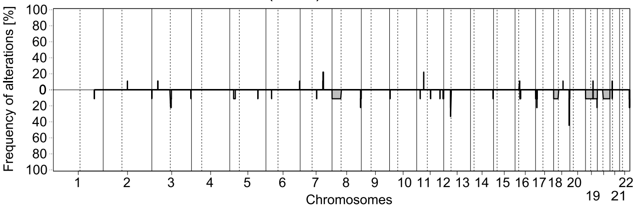

Gastrointestinal stromal tumour (n = 48)

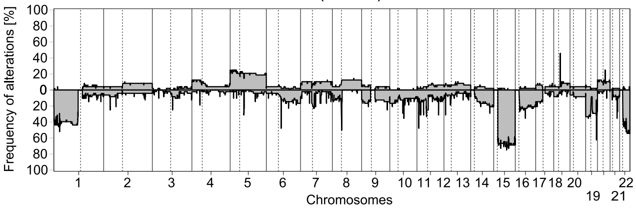

Infantile fibrosarcoma (n = 14)

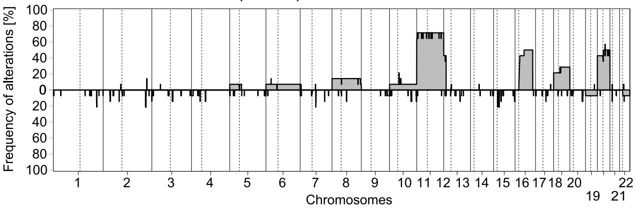

Inflammatory myofibroblastic tumour (n = 7)

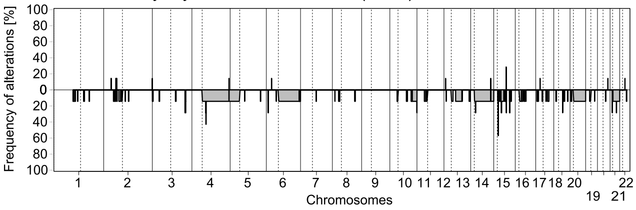

Kaposi sarcoma (n = 8)

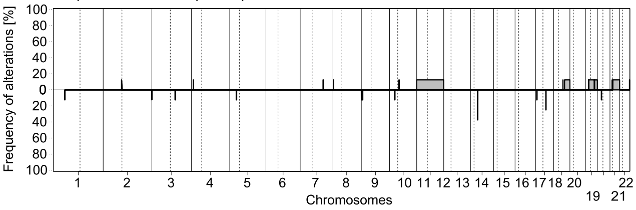

Langerhans cell histiocytosis (n = 11)

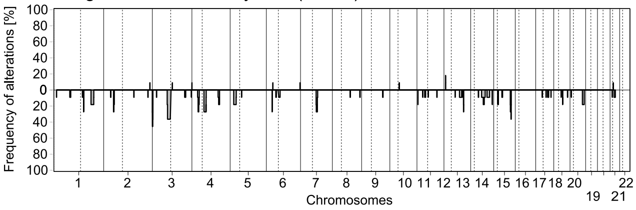

Low-grade fibromyxoid sarcoma (n = 8)

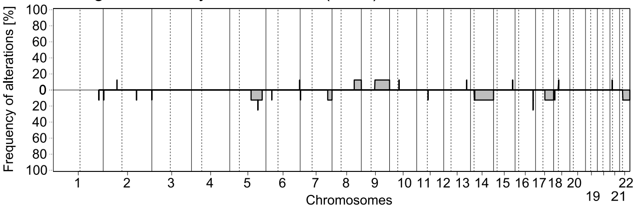

Lipoma (n = 10)

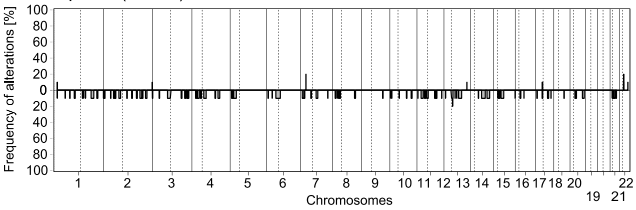

Leiomyoma (n = 7)

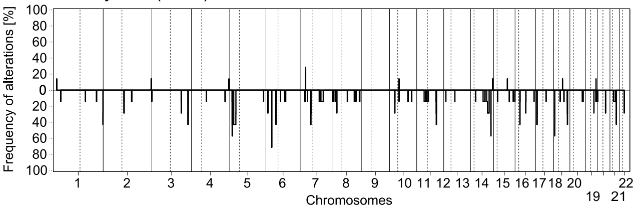

Leiomyosarcoma (n = 18)

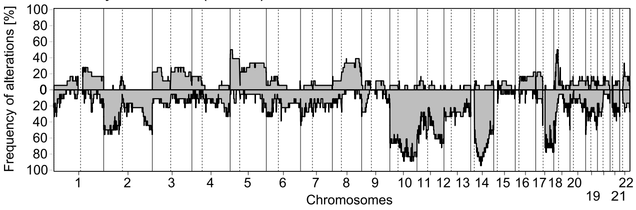

Melanoma - cutaneous (n = 12)

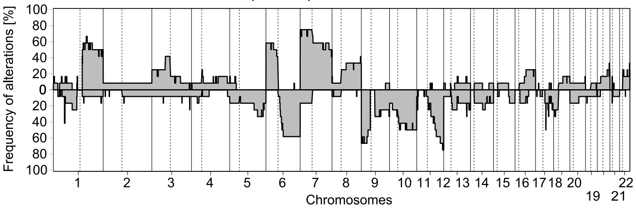

Myxoid liposarcoma (n = 31)

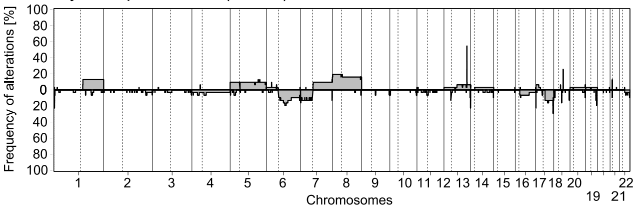

Myositis ossificans (n = 8)

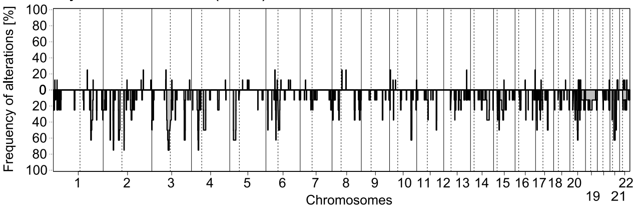

Supplementary Figure 5 - page 4

Myositis proliferans (n = 7)

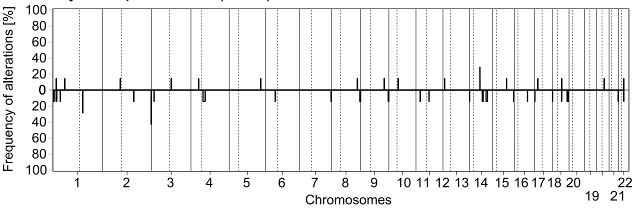

Malignant peripheral nerve sheath tumour (n = 26)

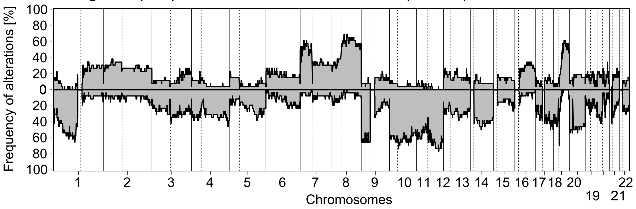

Malignant rhabdoid tumour (n = 17)

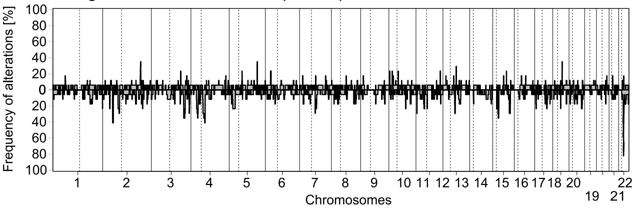

Nodular fasciitis (n = 10)

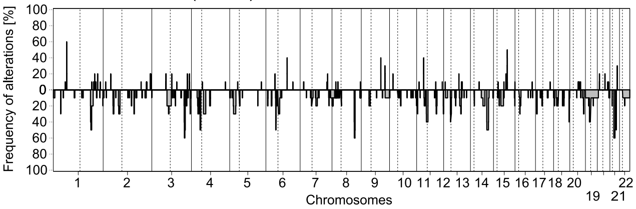

Neurofibroma (n = 9)

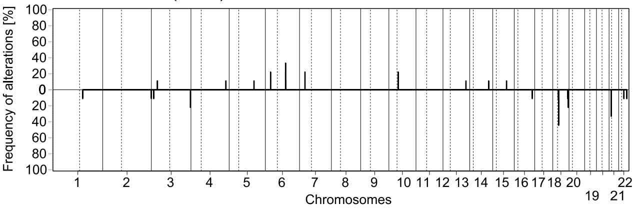

Neurofibroma - plexiform (n = 7)

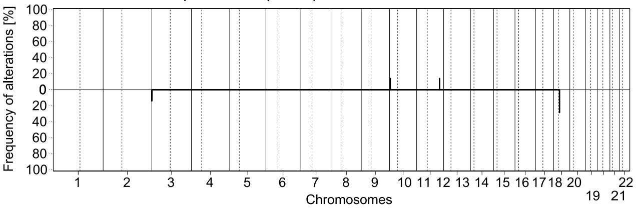

Osteoblastoma (n = 7)

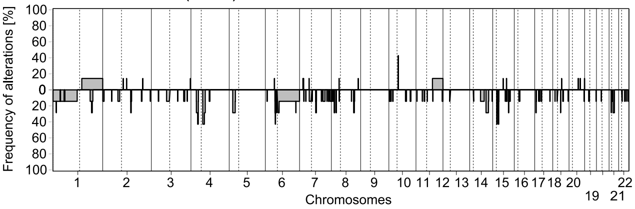

Ossifying fibromyxoid tumour (n = 13)

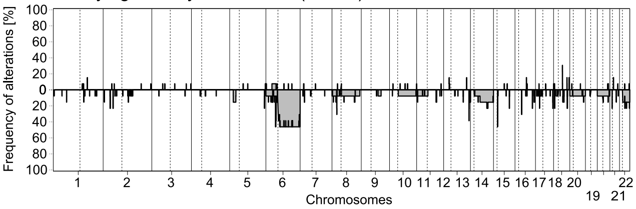

Osteosarcoma - conventional high-grade (n = 44)

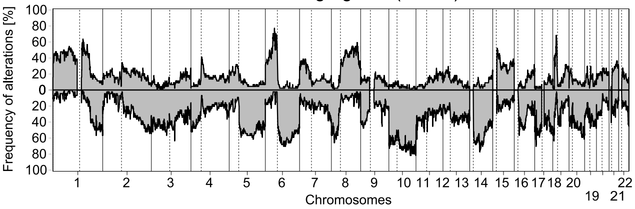

Rhabdomyosarcoma - alveolar (n = 36)

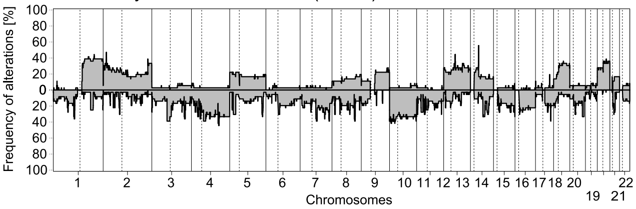

Rhabdomyosarcoma - embryonal (n = 28)

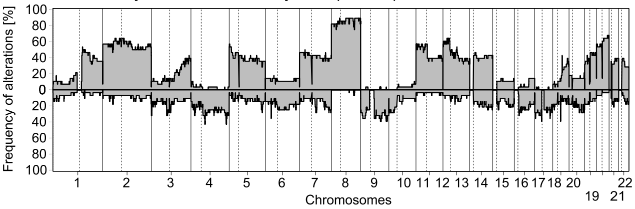

Rhabdomyosarcoma - MYOD1 (n = 7)

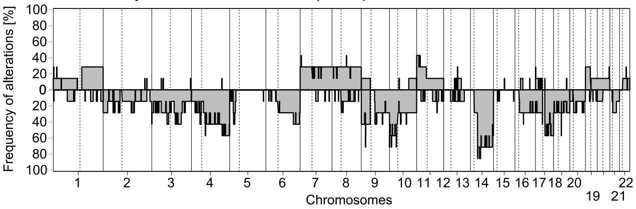

Sarcoma - MPNST-like (n = 7)

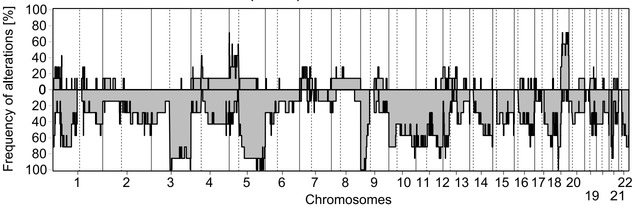

Sarcoma - RMS-like (n =17)

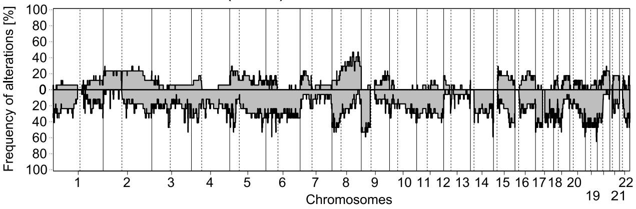

Supplementary Figure 5 - page 5

Small-, blue-, round-cell tumour - BCOR (n = 8)

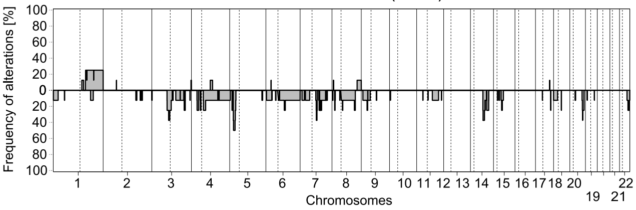

Small-, blue-, round-cell tumour - CIC (n = 11)

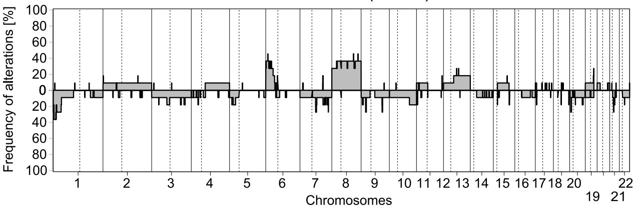

Squamous cell carcinoma - cutaneous (n = 10)

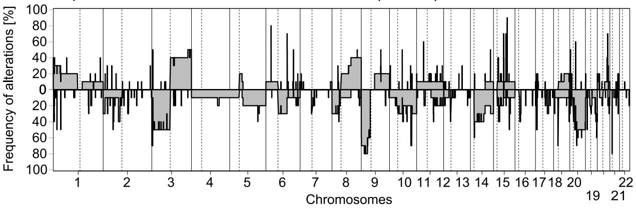

Sclerosing epithelioid fibrosarcoma (n = 7)

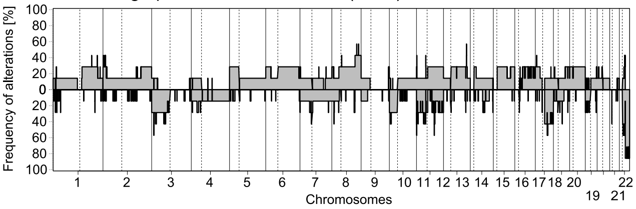

Solitary fibrous tumour (n = 26)

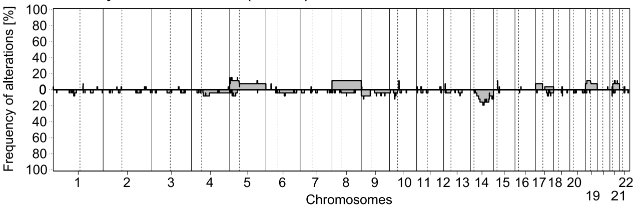

Schwannoma (n = 27)

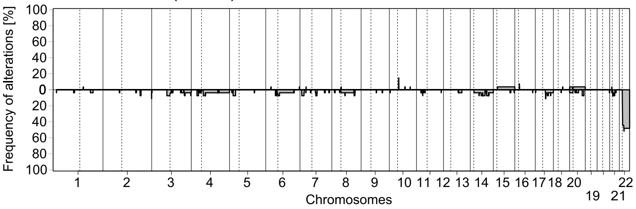

Synovial sarcoma (n = 39)

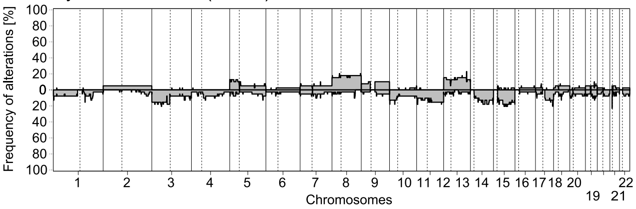

Undifferentiated sarcoma (n = 26)

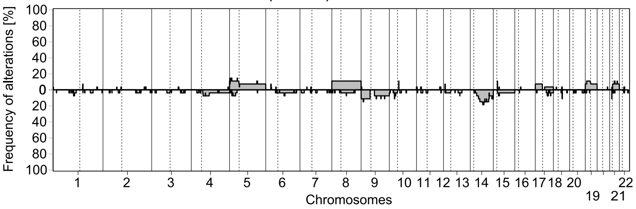

Well- / Dedifferentiated liposarcoma (n = 21)

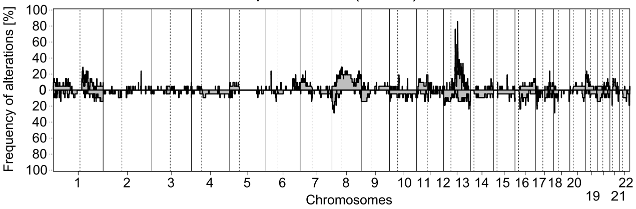

Supplementary Figure 6

Methylation profiling report

Supplier information

Sample identifier:	**168142**
Sentrix ID:	**203866300032_R02C01**
Material type:	**FFPE DNA**
Gender:	**female**
Supplier diagnosis:	**-**

**Automatic prediction**		
Array type:	**EPIC**	
Material type:	**FFPE DNA**	✔
Gender:	**female**	✔

Legend: ✔ OK ! Supplier information or prediction not available ✘ Warning, missmatch of prediction and supplier information

Sarcoma methylation classifier results (v12.2)

**Methylation classes (MCs with score >= 0.3)**	**Calibrated score**	**Interpretation**	
methylation class sarcoma (RMS-like)	0.99	match	✔

Legend: ✔ Match (score >= 0.9) ✘ No match (score < 0.9): possibly still relevant for low tumor content and low DNA quality cases. ● Match to MC family member (score >= 0.5)

Class descriptions

Methylation class sarcoma (RMS-like): The methylation class "sarcoma (RMS-like)" is based on tumours with the histological diagnosis of malignant CNS sarcoma / tumour not otherwise specified. Median age is 7 years (age range 0 to 76). The molecular hallmark of this class are DICER1 mutations (>90%), either somatic or inherited, often together with MAPK pathway alterations (>70%). Copy number profiles range from relatively flat to complex. Rhabdomyosarcoma of the genitourinary tract with DICER1 mutations may also assign to this methylation class. The name given here is provisional.

Copy number variation profile

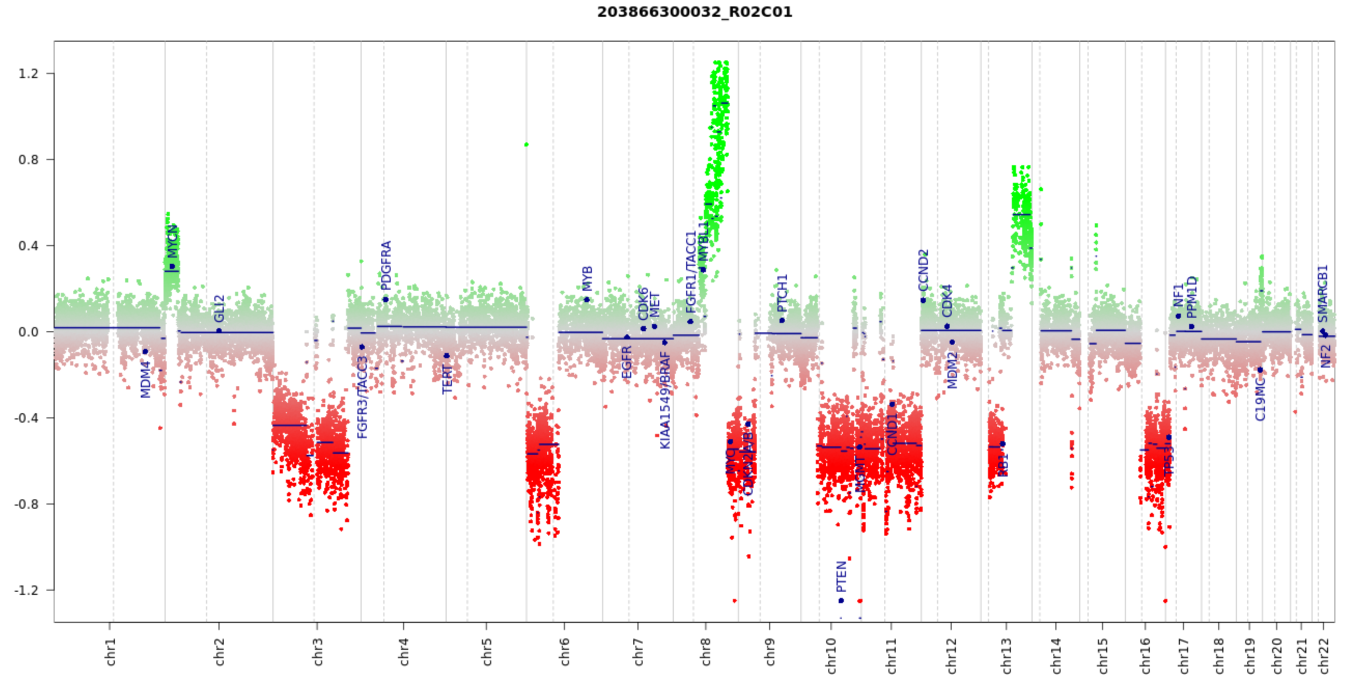

Depiction of chromosome 1 to 22 (and X/Y if automatic prediction was successful). Gains/amplifications represent positive, losses negative deviations from the baseline. 29 brain tumor relevant gene regions are highlighted for easier assessment.
(see Hovestadt & Zapatka, http://www.bioconductor.org/packages/devel/bioc/html/conumee.html)

Supplementary Figure 7

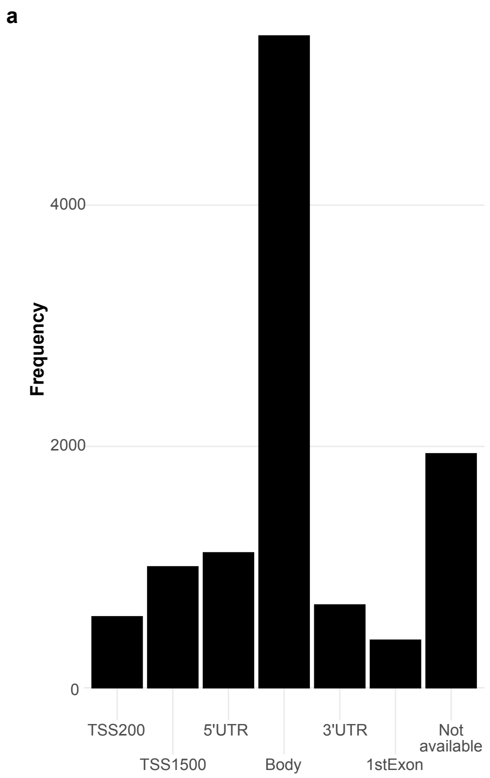

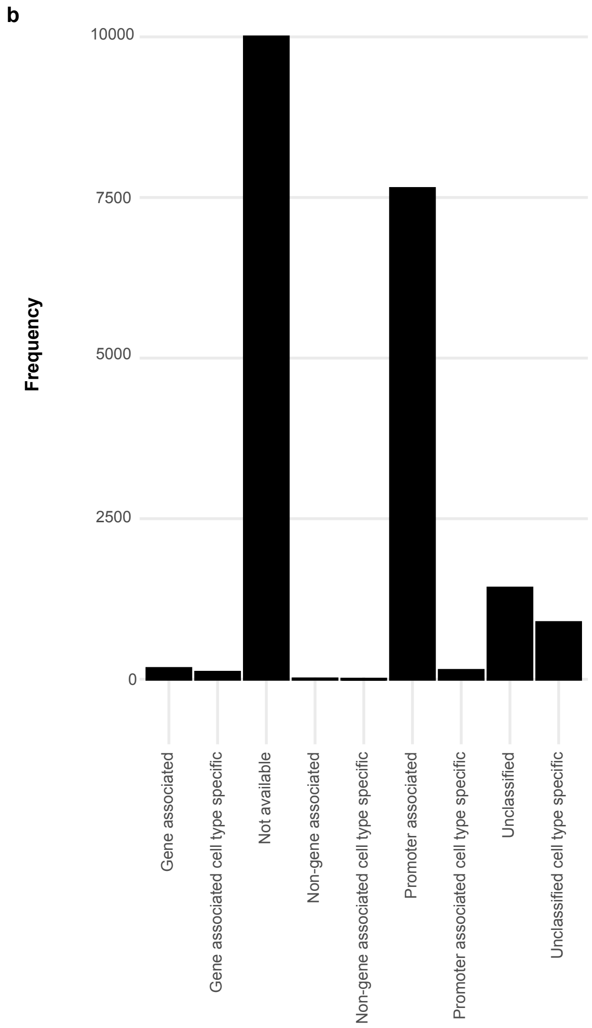

Supplementary Figure 8

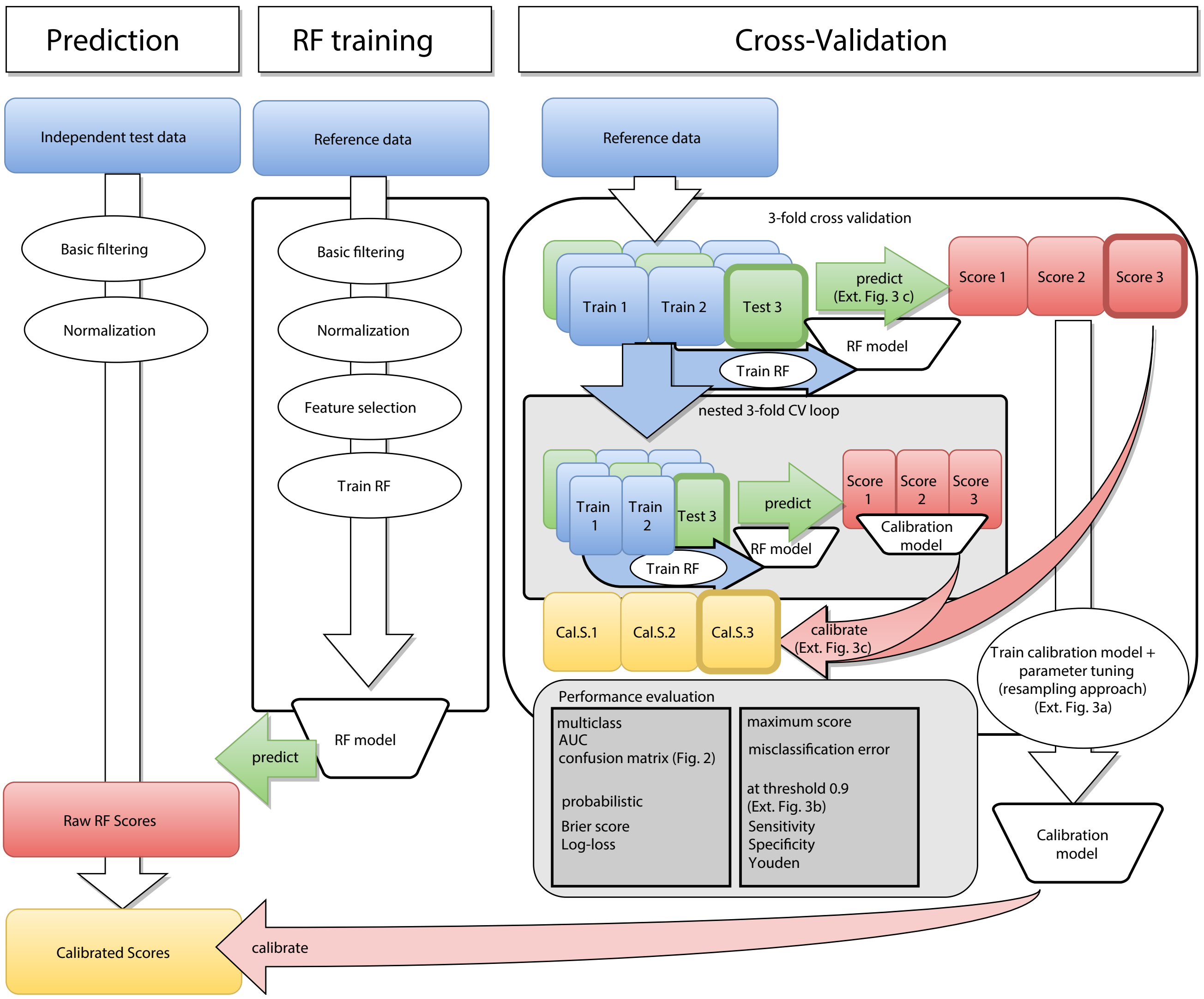

Description of Additional Supplementary Files

Supplementary Figure 1: Methylation class stability of the sarcoma classifier.

a) t-SNE position: t-SNE stability was assessed by performing separate t-SNE analyses of 500 datasets each generated by random down-sampling to 90% of the 1,077 cases. The class colours refer to Figure 1a. The depiction of the X and Y-axis positioning across the first 5 down-sampling iterations illustrates the close proximity of cases of the same class when perturbing the dataset. This indicates high group stability independent of the exact composition of the reference cohort. **b)** Pairwise correlation of X and Y coordinates for each case over the down sampling analysis demonstrates a very high correlation within classes (average correlation 0.9817) thereby indicating high stability of the t-SNE analysis.

Supplementary Figure 2: Unsupervised clustering is not biased by possible confounding factors.

t-SNE representations of the reference cohort annotated with the potentially relevant confounding factors do not indicate confounding effects on the unsupervised t-SNE analysis. The colour code is identical to Fig. 1a. **a)** reference classes according to Figure 1b; **b)** gender distribution; **c)** patient age at diagnosis; **d)** type of material used for DNA extraction; **e)** DNA methylation array type; **f)** tumour cell purity prediction.

Supplementary Figure 3: Threshold definition and implementation of calibrated classifier scores.

a) Illustration of the penalization parameter selection approach. The penalization parameter of the calibration models is selected such that at a fixed threshold of 0.9 the mean Youden index estimated over n = 500 bootstrap iterations is maximal. Error bars indicate the standard deviation (SD) of the Youden index estimated over n = 500 bootstrap iterations. **b)** Receiver

operating characteristic (ROC) curve for the calibrated maximum classifier methylation class family scores generated by cross-validation. The threshold 0.9 achieves a maximum specificity of 0.667 with a sensitivity of 0.963. Of note, the ROC curve illustrates the maximum score over all methylation classes. **c)** Overlaying density plots illustrating the distribution of cross-validated, raw and calibrated methylation class family classifier scores for correctly classified samples, depicted for each methylation class. The threshold 0.9 is highlighted by a red line.

Supplementary Figure 4: Classifier prediction for the validation cohort.

The colour indicates the relative frequency of samples with an initial diagnosis. The numbers in the heatmap denote the absolute number of samples. The abbreviations are identical to Figure 1a.

Supplementary Figure 5: Overview of cumulative copy number profiles of the 65 methylation classes.

Alterations above the zero-line indicate chromosomal gain, whereas alterations below the zero-line indicate chromosomal losses. The most frequent copy number variants are indicated in the methylation class descriptions (see Supplementary Data 2).

Supplementary Figure 6: Report example of the sarcoma classifier.

Sample website PDF report of a rare DICER1 associated CNS sarcoma matching with the methylation class Sarcoma (RMS-like).

Supplementary Figure 7: Distribution of CpG positions utilized by the sarcoma classifier.

The figure shows the distribution of the CpGs that are used by the classifier in relation to **a)** the functional gene subregion and **b)** their regulatory feature group.

Supplementary Figure 8: Diagram of the development stages of the random forest classifier.

The diagram shows how the classifier was trained and validated applying a 3x3 cross-validation. The nested cross-validation loop is needed to validate the score calibration.
