# Supplementary material for: Sarcoma classification by DNA methylation profiling
Source: Nat Commun. 2021 Jan 21;12:498. doi: 10.1038/s41467-020-20603-4 (PMC7819999; doi:10.1038/s41467-020-20603-4)
Supplement: Supplementary file 3 — Description of Additional Supplementary Files [file 41467_2020_20603_MOESM3_ESM.pdf]

Description of Additional Supplementary Files

File Name: Supplementary Data 1.

Description: Reference cohort. This table gives case-by-case details of the n=1077 biologically independent samples constituting the reference cohort including the Sentrix ID (idat), tissue source, clinical data, methylation class and technical specifications.

File Name: Supplementary Data 2.

Description: Overview of reference methylation class characteristics. This table gives an overview of the main characteristics of the methylation classes including full names of the methylation class, association of class with a methylation class family, number of cases per class, class age characteristics, male / female ratio, tumour localization, most frequent pathological diagnoses and a running text summarizing typical class features. Further, the Hex colour code of the reference classes used throughout this manuscript is provided.

File Name: Supplementary Data 3.

Description: Validation cohort. This table gives case-by-case details of the n=428 biologically independent samples constituting the prospective clinical cohort including information on the tissue source, clinical data, methylation class prediction, interpretation of classification and technical specifications.

File Name: Supplementary Data 4.

Description: Threefold cross-validation on the reference cohort. Threefold cross-validation of the Random Forest classifier incorporating information of n=1,077 biologically independent samples allocated to 65 methylation classes. Shown are the classification rates depending on raw and calibrated scores. The classification rates from the calibrated scores are illustrated in the heatmap in Figure 2.
