# Supplementary material for: Sarcoma classification by DNA methylation profiling
Source: Nat Commun. 2021 Jan 21;12:498. doi: 10.1038/s41467-020-20603-4 (PMC7819999; doi:10.1038/s41467-020-20603-4)
Supplement: Supplementary file 8 — Reporting Summary [file 41467_2020_20603_MOESM8_ESM.pdf]

nature research

Corresponding author(s): Andreas von Deimling ncomms-20-11551

Last updated by author(s): Nov 22, 2020

Reporting Summary

Nature Research wishes to improve the reproducibility of the work that we publish. This form provides structure for consistency and transparency in reporting. For further information on Nature Research policies, see our Editorial Policies and the Editorial Policy Checklist.

Statistics

For all statistical analyses, confirm that the following items are present in the figure legend, table legend, main text, or Methods section.

n/a	Confirmed	
☐	☒	The exact sample size (*n*) for each experimental group/condition, given as a discrete number and unit of measurement
☐	☒	A statement on whether measurements were taken from distinct samples or whether the same sample was measured repeatedly
☐	☒	The statistical test(s) used AND whether they are one- or two-sided<br>*Only common tests should be described solely by name; describe more complex techniques in the Methods section.*
☐	☒	A description of all covariates tested
☐	☒	A description of any assumptions or corrections, such as tests of normality and adjustment for multiple comparisons
☐	☒	A full description of the statistical parameters including central tendency (e.g. means) or other basic estimates (e.g. regression coefficient) AND variation (e.g. standard deviation) or associated estimates of uncertainty (e.g. confidence intervals)
☒	☐	For null hypothesis testing, the test statistic (e.g. *F*, *t*, *r*) with confidence intervals, effect sizes, degrees of freedom and *P* value noted<br>*Give P values as exact values whenever suitable.*
☒	☐	For Bayesian analysis, information on the choice of priors and Markov chain Monte Carlo settings
☐	☒	For hierarchical and complex designs, identification of the appropriate level for tests and full reporting of outcomes
☐	☒	Estimates of effect sizes (e.g. Cohen's *d*, Pearson's *r*), indicating how they were calculated

Our web collection on statistics for biologists contains articles on many of the points above.

Software and code

Policy information about availability of computer code

Data collection	R version 3.4.4 (R Development Core Team, 2019)
Data analysis	minfi Bioconductor package version 1.24.0, limma package version 3.34.5, Rtsne version 0.13, R package randomForest version 4.6-12, R package glmnet version 2.0-18, R-package conumee, R-package RF_Purify, also see https://github.com/mwsill/mnp_training

For manuscripts utilizing custom algorithms or software that are central to the research but not yet described in published literature, software must be made available to editors and reviewers. We strongly encourage code deposition in a community repository (e.g. GitHub). See the Nature Research guidelines for submitting code & software for further information.

Data

Policy information about availability of data

All manuscripts must include a data availability statement. This statement should provide the following information, where applicable:

- Accession codes, unique identifiers, or web links for publicly available datasets
- A list of figures that have associated raw data
- A description of any restrictions on data availability

Methylation data required for building the sarcoma classifier (reference set) were deposited at the public repository Gene Expression Omnibus (GEO; http://www.ncbi.nlm.nih.gov/geo) under the accession number (GSE140686). Supplementary Table 1 indicates the IDAT file names for each case. The remaining data are available within the Article, Supplementary Information or available from the authors upon request.

Field-specific reporting

Please select the one below that is the best fit for your research. If you are not sure, read the appropriate sections before making your selection.

☒ Life sciences ☐ Behavioural & social sciences ☐ Ecological, evolutionary & environmental sciences

For a reference copy of the document with all sections, see nature.com/documents/nr-reporting-summary-flat.pdf

Life sciences study design

All studies must disclose on these points even when the disclosure is negative.

Sample size	Classifier methylation classes need at least 7 cases (pre-established, see the brain tumour classifier "Capper et al., Nature 2018)
Data exclusions	No data sets were excluded.
Replication	The separation of samples into the defined DNA methylation classes was reliably reproduced by iterative random downsampling of the reference cohort.
Randomization	The methylation classifier reference cohort was compiled to recapitulate the entities established in the WHO classification of soft tissue and bone tumours. No randomization was performed.
Blinding	Blinding was not relevant to this study, because the classifier prediction result is an objective measure, which was compared with the institutional diagnosis.

Reporting for specific materials, systems and methods

We require information from authors about some types of materials, experimental systems and methods used in many studies. Here, indicate whether each material, system or method listed is relevant to your study. If you are not sure if a list item applies to your research, read the appropriate section before selecting a response.

Materials & experimental systems

n/a	Involved in the study	
☒	☐	Antibodies
☒	☐	Eukaryotic cell lines
☒	☐	Palaeontology and archaeology
☒	☐	Animals and other organisms
☐	☒	Human research participants
☒	☐	Clinical data
☒	☐	Dual use research of concern

Methods

n/a	Involved in the study	
☒	☐	ChIP-seq
☒	☐	Flow cytometry
☒	☐	MRI-based neuroimaging

Human research participants

Policy information about studies involving human research participants

Population characteristics	Reference cohort: 1077 cases, 502 females + 575 males<br>Validation cohort: 428 cases, 195 females + 233 males
Recruitment	Reference cohort: national and international sarcoma (reference) centres<br>Validation cohort: MASTER, INFORM, MNP2.0, PTT2.0 trials
Ethics oversight	This study has been performed in accordance with the Declaration of Helsinki.

Note that full information on the approval of the study protocol must also be provided in the manuscript.
